# Methylphenidate as a cognitive enhancer in healthy young people

**DOI:** 10.1590/S1980-5764-2016DN1002009

**Published:** 2016

**Authors:** Silmara Batistela, Orlando Francisco Amodeo Bueno, Leonardo José Vaz, José Carlos Fernandes Galduróz

**Affiliations:** 1 MS, Departamento de Psicobiologia - Universidade Federal de São Paulo, São Paulo SP, Brazil.; 2 PhD,Departamento de Psicobiologia - Universidade Federal de São Paulo, São Paulo SP, Brazil.

**Keywords:** central nervous system-stimulating drugs, ethics, nootropic drugs, performance enhancing substances, cognition

## Abstract

The so-called cognitive enhancers have been widely and increasingly used by healthy individuals who seek improvements in cognitive performance despite having no pathologies. One drug used for this purpose is methylphenidate, a first-line drug for the treatment of attention deficit hyperactivity disorder (ADHD).

**Objective:**

The aim of the present study was to test the effect of acute administration of varying doses of methylphenidate (10 mg, 20 mg, 40 mg and placebo) on a wide range of cognitive functions in healthy young people.

**Methods:**

A total of 36 young university students and graduates participated in the study. The participants underwent tests of attention and of episodic, and working memory.

**Results:**

No differences in performance were observed on any of the tests. There was a dose-dependent (40 mg > placebo) effect on self-reported wellbeing.

**Conclusions:**

According to the recent literature, psychostimulant medications, such as methylphenidate, improve performance when cognitive processes are below an optimal level, which was not the case for the subjects of the present study. We suggest the impression that methylphenidate enhances cognitive performance in healthy young people, justifying its use, may be due to improvements in subjective wellbeing promoted by the drug.

## RESUMO

Os chamados ampliadores cognitivos têm sido ampla e crescentemente utilizados por indivíduos saudáveis, que apesar de não apresentarem nenhum tipo de patologia, buscam por melhoras no desempenho cognitivo. Um fármaco utilizado para este fim é o metilfenidato, droga de primeira escolha para tratamento do transtorno de déficit de atenção e hiperatividade (TDAH).

**Objetivo:**

O presente estudo teve como objetivo verificar o efeito da administração aguda de diferentes doses de metilfenidato (10, 20 e 40 mg e placebo) sobre uma ampla gama de funções cognitivas em jovens saudáveis.

**Métodos:**

Participaram do estudo 36 jovens universitários ou graduados, tendo sido realizados testes de atenção, memória operacional, episódica.

**Resultados:**

Não foram observadas diferenças no desempenho dos sujeitos em nenhum dos testes. Houve efeito na auto-avaliação de bem estar, sendo este efeito dose dependente (40 mg > placebo).

**Conclusão:**

De acordo com a literatura recente, medicações psicoestimulantes, como o metilfenidato, produzem melhoras no desempenho quando os processos cognitivos estão abaixo de um nível ótimo, o que não era o caso dos sujeitos do presente estudo. Sugerimos que a impressão de que o metilfenidato melhora o desempenho cognitivo em pessoas jovens e saudáveis se deve ao seu efeito subjetivo de bem-estar.

## INTRODUCTION

Cognitive enhancers are drugs prescribed to improve cognitive performance in elderly patients and those with dementia or to promote better quality of life in patients with neuropsychiatric disorders or brain trauma.^1^ ^-^ ^3^ However, the administration of these drugs has gone beyond clinical indications, being increasingly used by healthy individuals seeking to improve their cognitive, emotional, and motivational functioning.^4^ ^-^ ^6^ Students use stimulant medications to improve academic performance, specifically by increasing levels of concentration and organization, and remaining awake longer for studying.^6^ ^,^ ^7^ Shift workers who perform functions at night (e.g., drivers and pilots) also seek to improve their cognitive performance by using cognitive enhancers, giving rise to situations that warrant careful attention.^1^ ^-^ ^3^

The use of drugs as cognitive enhancers is a controversial issue that involves various points of view and has enormous economic, ethical, and scientific implications.^1^ Some authors^8^ ^-^ ^10^ support the use of such drugs, arguing that cognitive enhancers are just another way of improving mental performance, and if they were really effective and safe, would provide great benefits for individuals and society. Chatterjee (2009)^10^ argues that non-therapeutic use of cognitive enhancers is premature because the efficacy and risk of these drugs in healthy individuals needs considerably more investigation. 

Additionally, there are ethical questions involved. Young people are pressured to exhibit good cognitive performance at work and in their studies. This pressure is amplified by the competitiveness of modern life, which expects ever more improvement and faster results.^2^

Administering these drugs in healthy young people should be evaluated carefully. If these drugs show positive effects in healthy individuals, questions about the potential effects of using cognitive enhancers, including the ethical, legal, and social implications, warrant urgent attention.^1^ ^,^ ^5^ ^,^ ^11^ Should there be no evidence of beneficial effects in healthy population, healthy individuals who are already disposed to accept the risks of consuming such drugs (based on their non-empirically proven benefits) should be made aware of this.^5^ ^,^ ^11^

Psychostimulant drugs with catecholaminergic action, such as amphetamine and methylphenidate, are among the substances most commonly used by individuals seeking to extend their capacities for alertness and cognition.^4^ ^,^ ^6^ Methylphenidate blocks the reuptake of dopamine and noradrenaline by blocking the transporters of these neurotransmitters, which leads to higher levels in the synaptic cleft.^6^ ^,^ ^12^ ^-^ ^14^

The dosage of methylphenidate is one of the factors that can determine the presence or absence of the cognitive effect. It is generally assumed that the pharmacokinetic properties of methylphenidate vary between individuals, as do the doses necessary to obtain clinical effects (0.1 to 1 mg/kg).^12^

Considering the growing and widespread use of methylphenidate as a cognitive enhancer and its uncertain effects in healthy young people, this study sought to verify the effect of acute administration of methylphenidate on memory, attention, and executive functions in healthy young people. The present study differs from previous studies in that it used a comprehensive battery of neuropsychological tasks to evaluate a broad range of drug doses on various cognitive functions. 

## METHODS

Subjects. Thirty-six (from 61 previously recruited) healthy male subjects aged between 18 and 30 years, with a minimum of 11 years of schooling, and Portuguese as their first language were included. All of the participants were informed of the objectives and procedures of the experiment and signed a free and informed consent form. The study was approved by the Research Ethic Committee of the Universidade Federal de São Paulo (CEP 0364/09).

Exclusion criteria were a body mass index lower than 17 or higher than 29.99; endocrine or heart diseases; sleep, neurological or psychiatric disorders; hearing or vision problems; signs of anxiety and/or depression; dependence on drugs of abuse; and therapy with psychotropic drugs. The following instruments were used: the State-Trait Anxiety Inventory (STAI),^15^ Beck Depression Inventory (BDI);^16^ ASRS-18 ADHD scale;^17^ and a semi-structured questionnaire. Clinical exams were performed: complete blood count, TSH, T3 and T4 hormones for thyroid function, and electrocardiogram. 

Three of the 25 subjects excluded showed signs of depression (BDI score greater than 20), three were excluded for changes on electrocardiogram, one for reported drug abuse, one for reported history of epilepsy, one for reported liver dysfunction, one for reported treatment of anxiety, one for reported treatment of depression, one for higher than expected erythrocyte levels, two for thyroid dysfunction, and one for signs of ADHD (score greater than 25 on ASRS); five subjects withdrew from the experiment after screening with no explanation. In the experimental phase, one participant was excluded due to sleep deprivation and four were excluded for scoring below average on the Raven, Vocabulary, and Cube tests. 

Procedure. The included subjects were instructed not to ingest alcohol or any psychoactive substance for 24 hours before and after the experiment and to get a good night's sleep and eat a light meal before the experiment. 

The experiment was double-blind, with a single orally-administered dose. Identical capsules containing 10 mg, 20 mg, or 40 mg of methylphenidate (Ritalin(r)) or placebo (starch) were administered to subjects who had been randomly assigned to each group. The capsule was ingested 90 minutes prior to commencement of the battery of tests so that its peak of absorption would be reached. Subjects were evaluated individually (between 11:00a.m. and 12:30p.m.) in a single session lasting approximately 90 minutes. 

The experimental session began by applying the instruments for estimating IQ (WAIS-R and Raven), followed by the Bodily Symptom Scale - BSS (modified by Greenwood et al., 1975),^18^ and ingesting the capsule. Subjects then waited for 90 minutes, during which time they remained comfortably seated in a room and could watch TV or read. At the end of the experiment, the subjects again completed the BSS scale. 

The Analogue Bodily Symptom Scale (BSS): This consists of 18 analogue scales that correspond to different bodily symptoms. The subjects must mark the appropriate point on the scale according to how they are feeling at the time. 

*Battery of tests.* Visual Attention Test (TAVIS 3):^19^ Selective-attention (to focus and select a particular stimulus while ignoring others), divided attention (to simultaneously process and respond to different stimuli from multiple sources), and sustained attention (to respond to a specific target-stimulus over a prolonged period). 

*Digit Span - Forward:* *^20^* Forward: temporary storage of acoustic information. Backward: to maintain and manipulate acoustic information. 

*^_Corsi Blocks: (computerized version adapted from Miyake, 2000): 21_^* Forward: temporary storage of visual and spatial information. Backward: to maintain and manipulate visual and spatial information.

*Operation Span (OSPAN)* *^22^* *task for words:* Episodic buffer, subcomponent for temporary storage that relates the contents of working memory to content already stored in long-term memory. 

*Stroop Test:* *^20^* Subcomponents of executive functions: processing speed, inhibition and monitoring. 

*Random Number Generation (RNG):* *^23^* Subcomponents of executive functions: maintenance of the set, adoption strategies for selection of appropriate responses and inhibition of inappropriate responses, monitoring, and modifying or alternating the strategies employed.

*Zoo Test:* *^24^* Planning domain of executive functioning. 

*Letter-memory:* *^21^* Updating domain of executive functioning. 

*Trail A and Trail B:* *^20^* Visual attention, mental and motor flexibility (alternation of executive functioning). 

*Logical Memory:* *^25^* *^,^* *^26^* Evaluates episodic memory.

Statistical analysis. Descriptive analyses (mean and standard deviation) were performed for all the variables, which were also tested for normality (K-S test) and homogeneity (Levene test). A one-way ANOVA model was used for the normally distributed variables. The Tukey-HSD post-hoc test was used when a difference between the group means was detected. The Kruskal-Wallis test was used for the non-normally distributed variables. The level of significance adopted was p < 0.05.

## RESULTS

Table 1 shows the demographic data for the study sample and their performance on the IQ tasks (raw data). No significant difference was detected.

**Table 1:** Demographic measures, raw scores on IQ tests, and depression, anxiety, and ADHD scores.

Variables	Methylphenidate			F	P	
	Placebo	10 mg	20 mg	40 mg		
N	9	9	9	9		
Age	22.22 ± 2.59	22.33 ± 2.69	23.67 ± 2.60	22.56 ± 2.60	0.57	0.63
Education	14.11 ± 2.71	13.56 ± 1.51	14.78 ± 2.68	13.78 ± 1.86	0.50	0.68
Raven	53.00 ± 5.61	52.44 ± 3.64	54.25 ± 3.01	55.11 ± 3.37	0.78	0.51
Cubes	52.22 ± 9.85	54.22 ± 9.24	56.00 ± 6.71	59.22 ± 5.21	1.24	0.30
Vocabulary	43.00 ± 3.20	43.22 ± 5.14	40.56 ± 4.07	44.78 ± 3.42	1.68	0.18
Beck	3.88 ± 3.01	4.11 ± 3.48	2.88 ± 3.4	5.33 ± 4.00	0.74	0.53
*STAI	31.66 ± 7.21	29.77 ± 7.12	29.5 ± 4.4	34.87 ± 10.98	0.83	0.48
**ASRS	17.22 ± 4.73	22.66 ± 7.88	22.11 ± 9.38	24.85 ± 7.96	1.46	0.24

*STAI: Scale for anxiety (state); **ASRS: Scale for ADHD.

On the selective and divided attention tasks, no differences between groups were detected on the measures of total correct responses, omissions, action or reaction time. On the sustained attention task, a ceiling effect was observed for all subjects. There were also no differences in the measures of action and reaction time (Table 2). 

**Table 2:** Correct-answers, omission, action and reaction time measures on tests for selective, divided and sustained attention.

	Methylphenidate				F	P
	Placebo	10 mg	20 mg	40 mg		
Selective attention						
Correct	19.44 ± 3.81	21.44 ± 2.12	20.11 ± 2.47	20.00 ± 3.12	0.74	0.53
Omission	3.88 ± 3.75	1.66 ± 2.44	3.11 ± 2.57	3.77 ± 3.89	0.89	0.45
Action	1.88 ± 1.9	1.33 ± 1.41	1.11 ± 1.26	1.66 ± 1.93	0.39	0.75
Reaction time	0.461 ± 0.03	0.456 ± 0.02	0.468 ± 0.02	0.467 ± 0.04	0.26	0.84
Divided attention						
Correct	21.55 ± 0.72	21.44 ± 0.72	21.88 ± 0.33	21.77 ± 0.44	2.95 (H)	0.39
Omission	1.66 ± 0.86	1.66 ± 0.7	1.55 ± 0.52	1.44 ± 0.52	0.22	0.87
Action	1.55 ± 0.88	2.22 ± 1.48	1.22 ± 0.66	1.44 ± 1.13	1.42	0.25
Reaction time	0.620 ± 0.08	0.627 ± 0.1	0.599 ± 0.05	0.590 ± 0.06	0.43	0.73
Sustained attention						
Correct	64 ± 0	64 ± 0	64 ± 0	64 ± 0		
Omission	0 ± 0	0 ± 0	0 ± 0	0 ± 0		
Action	0.11 ± 0.33	0.11 ± 0.33	0.44 ± 1.01	0.11 ± 0.33	0.73	0.53
Reaction time	0.349 ± 0.04	0.347 ± 0.08	0.343 ± 0.03	0.331 ± 0.05	0.16	0.92

There was no significant difference between the groups on the Corsi or Digit span forward and backward tests (Table 3). 

**Table 3:** Means and standard deviations of digit span forwards (phonological loop) and backwards (central executive/executive functions); letter memory (executive function); Stroop 1, Stroop 2, and Stroop 3 (central executive/executive function); Trail A and Trail B (central executive/executive functions); Corsi forwards (visuospatial sketchpad) and backwards (central executive/executive function) from the measures for the OSPAN test (visuospatial sketchpad); and random number generation test (central executive/executive function).

Tests	Methylphenidate				F/H	P
	Placebo	10 mg	20 mg	40 mg		
Digit Span Forwards	6.33 ± 1.32	5.77 ± 1.09	7.11 ± 1.26	7.22 ± 0.66	2.58	0.07
Digit Span Backwards	6.11 ± 1.61	5.11 ± 1.26	5.88 ± 1.05	6.33 ± 1.11	1.54	0.25
Letter Memory	9.33 ± 3.31	9.33 ± 3.93	8.55 ± 2.18	8.55 ± 2.55	0.19	0.90
Stroop 1	47.55 ± 11.86	50.44 ± 10.91	46.55 ± 9.68	47.55 ± 9	0.23	0.87
Stroop 2	54.33 ± 12.45	58,33 ± 12.12	52 ± 9.88	54.11 ± 10.75	0.48	0.69
Stroop 3	71 ± 14.3	76.77 ± 6.27	66.77 ± 13.76	68.11 ± 15.92	0.89	0.45
Trail A	28.77 ± 9.07	25.88 ± 4.13	25.88 ± 4.13	28.5 ± 12.46	0.31	0.81
Trail B	48.77 ± 14.83	59.88 ± 19.71	60 ± 35.97	58 ± 20.74	0.43	0.72
Corsi Forwards	6.88 ± 1.69	6.44 ± 1.13	6.55 ± 0.88	6.33 ± 1.11	0.33	0.79
Corsi Backwards	6 ± 1.41	6.33 ± 1.5	5.77 ± 0.83	6.55 ± 1.5	0.59	0.62
OSPAN - Sums	0.99 ± 0.01	0.98 ± 0.01	0.98 ± 0.02	0.98 ± 0.01	0.36	0.78
OSPAN - Serial Recall	0.56 ± 0.17	0.58 ± 0.19	0.67 ± 0.16	0.59 ± 0.18	0.63	0.59
OSPAN - Free Recall	0.71 ± 0.1	0.75 ± 0.11	0.78 ± 0.11	0.73 ± 0.1	0.67	0.57
RNG - Evans Index	0.32 ± 0.04	0.31 ± 0.02	0.33 ± 0.02	0.31 ± 0.03	1.03	0.38
RNG - Coupon	17.00 ± 4.96	18.77 ± 7.71	17.53 ± 5.15	17.74 ± 2.31	0.17	0.91
RNG - TPI*	98.63 ± 9.71	94.65 ± 13.08	95.91 ± 8.2	98.36 ± 7.89	0.33	0.79
RNG - Runs	0.89 ± 0.27	0.89 ± 0.28	0.81 ± 0.2	0.87 ± 0.19	0.19	0.90
RNG - Average	8.78 ± 0.14	8.69 ± 0.23	8.68 ± 0.41	8.73 ± 0.11	0.28	0.83
RNG - Preferred	15.11 ± 1.83	15.00 ± 1.8	14.88 ± 1.61	13.22 ± 2.86	1.65	0.19
RNG - Less Preferred	7.22 ± 1.71	6.67 ± 1.58	7.11 ± 2.31	8.00 ± 2.29	0.68	0.56
RNG - Errors	2.66 ± 3.39	4.55 ± 5.61	3.11 ± 4.25	4.77 ± 4.79	0.46	0.70
Zoo - Score (I**)	6.33 ± 2.5	6.88 ± 2.2	7.44 ± 1.33	6.44 ± 2.45	0.92	0.82
Zoo - Planning (I**)	73.77 ± 39.19	100 ± 65.48	50 ± 25.83	121.66 ± 129.07	3.52	0.31
Zoo - Total Time (I**)	145 ± 105.67	138.44 ± 71.45	82 ± 33.99	163.11 ± 137.61	4.32	0.22
Zoo - Score (II^#)^	8.00 ± 0	8.00 ± 0	7.88 ± 0.33	8.00 ± 0	3.00	0.39
Zoo - Time (II^#)^	31.66 ± 9.68	33.88 ± 14.47	32.77 ± 12.98	35 ± 14.71	0,29	0.96

*TPI: Turning Point Index; **I: First part, with high cognitive demand; ^#^II: Second part, with low cognitive demand.

On the OSPAN test, the groups performed similarly on all measures assessed (Table 3).

There were no significant differences between the groups on any of the tests of randomness in number generation (RNG) (Table 3). 

There were no significant differences between the groups on the other tests used to assess central executive functions. The groups exhibited similar behavior on the Letter memory, Stroop and Trail tests (Table 3).

The groups did not differ in immediate or delayed recall on the logical memory task (episodic memory) (Table 4). 

**Table 4:** Means and standard deviations for immediate and delayed recalls on test of logical memory.

Logical Memory	Methylphenidate				F	P
	Placebo	10 mg	20 mg	40 mg		
Immediate Recall	31.44 ± 2.40	29.00 ± 6.36	28.11 ± 5.97	26.44 ± 5.32	1.42	0.25
Delayed Recall	27.78 ± 3.03	26.22 ± 6.76	25.33 ± 8.75	26.00 ± 5.45	0.23	0.86

BSS scale of somatic symptoms. There was a dose-dependent effect on the BSS. The scales were completed by the subjects prior to administration of the drug (pre-treatment) and again at the end of the experiment, after administering the neuropsychological tests (post-treatment).

Improved "general wellbeing" was detected on the post-treatment assessment. The groups that received 20 mg and 40 mg of methylphenidate reported a better subjective feeling of wellbeing than the placebo group (U = 17.50 with p < 0.03 and U = 13.00 with p < 0.01, respectively). The same effect was not observed in the group that received 10 mg of methylphenidate (U = 26.51 with p = 0.21). There were no differences between the groups on the other scale items (Table 5).

**Table 5:** Means and standard deviations for items on self-evaluation scale performed before experiment (pre-treatment) and after the battery of tests (post-treatment).

Bodily Symptoms Scale (BSS)		Methylphenidate				F/H	P
		Placebo	10 mg	20 mg	40 mg		
Pre-Treatment	Physical exhaustion	29.46 ± 22.57	20.57 ± 23.52	38.63 ± 29.62	29.53 ± 30.69	0.67	0.57
	Headache	5.13 ± 11	2.3 ± 3.54	1.42 ± 4.26	4.81 ± 11.76	0.41	0.74
	Dizziness	10.14 ± 21.05	0.86 ± 1.83	10.82 ± 27.37	7.26 ± 13.67	0.53	0.65
	Trembling	8.25 ± 15.13	6.89 ± 12.42	3.6 ± 6.99	4.99 ± 9.45	0.29	0.83
	Weakness	9.15 ± 13.75	6.32 ± 10.37	0.86 ± 2.6	2.14 ± 3.74	5.05(H)	0.16
	Muscular Tension	19.5 ± 17.83	22.02 ± 21.65	6.71 ± 5.72	8.97 ± 20.88	1.64	0.19
	Nausea	1.36 ± 4.08	1.29 ± 2.33	6.41 ± 19.23	1 ± 2.2	1.31(H)	0.72
	Dry mouth	51.05 ± 26.37	54.64 ± 35,52	59.82 ± 29.98	67 ± 26.52	0.48	0.69
	Sweat	24.55 ± 31	21.1 ± 29.09	12.04 ± 17.27	11.83 ± 30.74	0.48	0.69
	Blurred vision	31.67 ± 31.07	23.53 ± 29.79	24.78 ± 25.95	27.72 ± 24.66	0.15	0.92
	Palpitation	4.57 ± 10.41	12.73 ± 16.29	13.32 ± 22.65	15.61 ± 29.06	0.48	0.69
	Difficulty breathing	1.57 ± 3.14	7 ± 14.35	2.56 ± 5.43	0.67 ± 1.36	0.87(H)	0.83
	Difficulty walking	3.13 ± 6.99	2.42 ± 3.26	5.27 ± 15.81	0.82 ± 1.63	0.39	0.75
	Agitation	25.62 ± 25.91	28.3 ± 27.58	28.86 ± 20.41	29.2 ± 27.89	0.03	0.99
	Motor Coordination	90.67 ± 15.29	91.41 ± 10.83	91.29 ± 20.58	96.72 ± 8.48	0.33	0.79
	Hearing	78.33 ± 39.23	95.38 ± 12.47	93.16 ± 20.51	99.13 ± 1.7	1.60(H)	0.65
	Difficulty speaking	17.01 ± 32.39	5.86 ± 7.92	1.99 ± 5.98	3.24 ± 7.13	3.31(H)	0,34
	General wellbeing	67,48 ± 38,46	92,23 ± 12,01	78,64 ± 32,56	90,88 ± 15,82	1,78(H)	0,61
Post-Treatment	Physical exhaustion	45.21 ± 16.82	17.02 ± 24.1	39.37 ± 33.5	33.45 ± 19.61	2.24	0.10
	Headache	13.67 ± 24.32	8.92 ± 19.51	3.41 ± 8.13	0.85 ± 1.81	3.36(H)	0.33
	Dizziness	9.98 ± 23.87	9.65 ± 21.08	11.12 ± 28.18	2.84 ± 4.98	0.27	0.84
	Trembling	10.11 ± 21.47	1,42 ± 1.75	3.16 ± 5.19	7.26 ± 15.26	0.12(H)	0.98
	Weakness	13.72 ± 22.27	7.99 ± 13.6	12.84 ± 28.32	6.55 ± 13.15	0.27	0.84
	Muscular Tension	12.71 ± 15.66	27.15 ± 24.33	11.14 ± 11.52	15.82 ± 21.45	2.64(H)	0.45
	Nausea	2.85 ± 4.39	2.14 ± 2.13	7.12 ± 21.36	0 ± 0	6.79(H)	0.07
	Dry mouth	66.6 ± 29.31	69.33 ± 19.68	62.25 ± 29.79	71.71 ± 28.45	0.20	0.89
	Sweating	9.02 ± 10.14	23.58 ± 33.77	19.97 ± 26.33	6.83 ± 20.51	3.65(H)	0.30
	Blurred vision	17.63 ± 23.88	31.58 ± 28.72	28.96 ± 21.68	28.71 ± 30.84	0.49	0.69
	Palpitation	11.34 ± 20.66	20.58 ± 24.2	5.98 ± 16.55	16.09 ± 21.64	0.80	0.49
	Difficulty breathing	7.12 ± 19.52	12.68 ± 22.29	4.7 ± 10.21	0 ± 0	6.74(H)	0.08
	Difficulty walking	9.98 ± 22.08	4.14 ± 7.18	6.12 ± 17.43	0.43 ± 1.3	0.67(H)	0.57
	Agitation	31.94 ± 29.54	30.44 ± 25.34	19.13 ± 20.93	30.53 ± 24.12	0.50	0.68
	Motor Coordination	80.43 ± 21.62	83.13 ± 21.35	80.63 ± 24.83	89.74 ± 19.14	0.35	0.78
	Hearing	92.42 ± 13.02	94.7 ± 6.78	86.03 ± 30.91	99.71 ± 0.86	4.10(H)	0.25
	Difficulty speaking	11.45 ± 17.93	8.43 ± 9.73	8.83 ± 26.01	0.56 ± 1.7	0.72	0.54
	General wellbeing	64.3 ± 34.8	88.1 ± 11.73	88.45 ± 22.06	93.72 ± 15.72	8.52(H)	0.03

## DISCUSSION

The objective of the present study was to verify the effects of methylphenidate on various cognitive functions in healthy young people. This drug, traditionally recognized as an effective treatment for improving ADHD symptoms,^1^ ^,^ ^14^ became widely used by healthy individuals as so-called cognitive enhancers,^5^ ^,^ ^9^ drugs that supposedly improve cognitive performance in individuals who take them despite having no neuropsychiatric, neurobehavioral, or neurological pathologies.^2^ ^,^ ^9^

Our results showed no effect of methylphenidate on cognitive functioning. It should be noted that we used various tests involving a large number of cognitive domains, as well as a wide range of clinical doses. However, methylphenidate improved the feeling of wellbeing, as assessed by the BSS scale. 

In the present study, attention was not altered after acute administration of the drug, possibly due to a ceiling effect reached by the participants, who obtained maximum (or near maximum) scores on the tests. In particular, the test of sustained attention lacked sensitivity for testing subjects with a high educational level, given the lack of variability between the groups. Elliot et al. (1997)^27^ also found no significant effect of acute administration of methylphenidate (20 and 40mg) on a test of sustained attention and verbal fluency. The authors did, however, observe improved performance on tasks dealing with visuo-spatial memory and planning, although these effects did not vary with dose. In the same study, a significant difference was also observed on the subjective scales answered by the subjects, and there were improvements in the measures of alertness and exhaustion (with the former increasing, and the latter decreasing). 

Tomasi et al. (2011)^28^ observed no cognitive differences compared to placebo on visual attention tasks and verbal working memory (n-back) in healthy subjects after acute administration of 20mg of methylphenidate. In the same study, functional magnetic resonance was simultaneously used while carrying out the tests. The methylphenidate group showed greater activation of the dorsal attention network (essential for top-down attention) than the placebo group and greater deactivation of the default mode network (which is active when not focusing on a specific task). These alterations were not associated with improved cognitive performance, however. The authors suggested that their results may reflect the brain activating neural reserve systems to maintain precision during resting conditions. This condition may be adversely affected by fatigue and sleep deprivation, which may explain why methylphenidate is particularly favorable for cognitive performance in these situations. In our study, the subjects were instructed to get a good night's sleep prior to the experiment and to be rested; based on the suggestion by Tomasi et al. (2011),^28^ where these conditions may have explained the absence of improvements. 

In addition to evaluating the visuo-spatial subcomponent, we applied tests to assess the other subcomponents of the working memory system, on which no differences were observed, in contrast with findings of a review conducted by Lissen et al. (2014).^29^

The improved cognitive performance after acute administration of methylphenidate may be dependent on variables such as the characteristics of the task and the baseline of the subjects.^14^ ^,^ ^30^ ^,^ ^31^ The participants in the present study were all students from renowned public universities and showed high scores on the IQ tests. According to Gamo et al. (2010),^32^ methylphenidate is particularly beneficial for subjects with a low capacity for working memory. 

Volkow et al. (2008)^33^ suggested that the effects of stimulants can be beneficial when neural resources are focused elsewhere or are adversely affected, but can have adverse effects when cerebral activity is already at an optimal level of focus. In their study, the authors ^33^ used PET to show that methylphenidate (20mg)given prior to a cognitive task significantly attenuated the cerebral metabolic increase necessary for performing the task and reduced activation of the regions involved in executive processes, orientation and attentional alertness compared to placebo. They concluded that methylphenidate reduced the use of attention resources required to achieve similar levels of performance compared to placebo. They also found that the subjects in whom the drug produced the greatest attenuations were those who had the lowest baseline cerebral metabolism and the greatest increases in cerebral metabolism when the task was performed after consuming placebo. The subjects who experienced smaller alterations after administration of methylphenidate showed the lowest activation during the task; they also experienced no drug effect on task performance. These authors^33^ concluded that these results support the notion that individuals who already possess an optimum level of cerebral resources would not benefit from use of the drug. 

Finger et al. (2003)^6^ stated there is no clear evidence that methylphenidate can enhance memory and learning, as we found in the present study, where no drug effect was observed in the test of episodic memory. A similar result was obtained by other researchers^34^ using associated-pair tasks and immediate and delayed recall of stories after intravenous administration of methylphenidate at doses of 0.1 and 0.25 mg/kg. At a higher dose (0.5 mg/kg), adverse effects on performance were observed. We did not observe any adverse effects of methylphenidate even at the highest (40mg/subject) orally administered dose. However, Finger et al. (2013)^6^ warned that healthy people who use stimulants as cognitive enhancers could experience impaired performance due to the euphoric state induced, preventing study for an exam, for example. 

According to Brignell et al. (2007),^35^ methylphenidate can promote feelings of wellbeing and elation. A similar change in the subjective "general wellbeing" measure, as assessed by the BSS scale after the 40mg dose, was the only significant effect found in the present study. Volkow et al. (2004)^13^ proposed that methylphenidate acts on the salience of the task, increasing motivation to perform tiresome and wearying tasks. Beyer et al. (2014)^14^ also commented on subjective feelings in healthy people who takes methylphenidate, suggesting that the supposed increase in performance might be more related to perceived improvement than with actual cognitive enhancement. According to the authors,^14^ sleep-deprived subjects who take this drug have a subjective sensation of stimulation and subsequently overestimate their performance.

To date, cognitive enhancers have been presented as a "double-edged sword": even if they could improve our lives, they could also be abused in an unethical manner.^11^

However, there are those who defend the use of cognitive enhancers by healthy individuals. According to Harris (2009),^10^ given that the drug is considered safe enough to be widely used by children and adults with ADHD, even over long periods of time, and given that no drug is free from side effects, its use by healthy individuals ought to be permitted and legalized. However, while use of cognitive enhancers in individuals with pathologies has many advantages for society, the situation is more complex for healthy individuals because the risks outweigh the benefits.^10^ ^,^ ^11^ ^,^ ^14^ Thus, some authors conclude that the argument for non-therapeutic uses of methylphenidate (and other cognitive enhancers) is premature and that the efficacy and risks in healthy individuals ought to be properly investigated.^6^ ^,^ ^10^ ^,^ ^11^ ^,^ ^14^ With respect to the safety of the drug, Greely et al. (2008)^8^ stated that the safety of cognitive enhancers is guaranteed only for therapeutic indications and is not necessarily valid for use by healthy subjects. Also, Norman and Berger (2008)^4^ stated that although some studies have shown potential improvements in learning and memory after pharmacological manipulations it is not yet clear to what degree such manipulations of cognitive processes may also lead to undesirable side effects. Therefore, despite the attraction of using cognitive enhancers, potential users should weigh up the pros and cons and bear in mind that these drugs were not originally developed for this purpose.^2^ ^,^ ^11^ The potential risks are not be justifiable given that no improvement was observed in any of the cognitive functions evaluated. 

In summary, the present study sought to test the effects of varying doses of methylphenidate on different cognitive functions. The results are in line with recent findings in the literature showing that improved performance is typically observed only when cognitive processes are below optimal levels, which was not the case for the subjects evaluated in the present study. We suggest that the impression of enhanced cognitive performance in healthy young people taking methylphenidate and justifying its use, may be due to improvements in subjective wellbeing promoted by the drug. 

Thus, greater caution and regulation of cognitive enhancers is suggested in healthy young people who take these medications despite having no clinical conditions indicating their use.

Based on our results, we believe that use of methylphenidate by healthy individuals is not justified. We further suggest that more studies be performed to confirm this view and that the results be widely publicized to increase the population's awareness of its lack of efficacy as a cognitive enhancer under these circumstances.
